# A novel cytosporone 3-Heptyl-4,6-dihydroxy-3*H*-isobenzofuran-1-one: synthesis; toxicological, apoptotic and immunomodulatory properties; and potentiation of mutagenic damage

**DOI:** 10.1186/s12885-015-1532-2

**Published:** 2015-07-31

**Authors:** Rodrigo Juliano Oliveira, Stephanie Dynczuki Navarro, Dênis Pires de Lima, Alisson Meza, João Renato Pesarini, Roberto da Silva Gomes, Caroline Bilhar Karaziack, Mariana de Oliveira Mauro, Andréa Luiza Cunha-Laura, Antônio Carlos Duenhas Monreal, Wanderson Romão, Valdemar Lacerda Júnior, Adilson Beatriz

**Affiliations:** 1 Centro de Estudos em Células Tronco, Terapia Celular e Genética Toxicológica – CeTroGen, Hospital Universitário “Maria Aparecida Pedrossian” – HUMAP, Empresa Brasileira de Serviços Hospitalares – EBSERH, Campo Grande, MS Brazil; 2 Programa de Mestrado em Farmácia, Centro de Ciências Biológicas e da Saúde – CCBS, Universidade Federal de Mato Grosso do Sul – UFMS, Campo Grande, MS Brazil; 3 Programa de Pós-graduação em Saúde e Desenvolvimento na Região Centro-Oeste, Faculdade de Medicina “Dr. Hélio Mandetta” – FAMED, Universidade Federal de Mato Grosso do Sul – UFMS, Campo Grande, MS Brazil; 4 Programa de Pós-graduação em Química, Instituto de Química, Universidade Federal de Mato Grosso do Sul – UFMS, Campo Grande, MS Brazil; 5 Faculdade de Ciências Exatas e Tecnologia – FACET, Universidade Federal da Grande Dourados – UFGD, Dourados, MS Brazil; 6 Programa de Doutorado em Biotecnologia e Biodiversidade – Rede Pró Centro-Oeste, Universidade Federal de Mato Grosso do Sul – UFMS, Campo Grande, MS Brazil; 7 Centro de Ciências Biológicas e da Saúde – CCBS, Universidade Federal de Mato Grosso do Sul – UFMS, Campo Grande, MS Brazil; 8 Departamento de Química, Universidade Federal do Espírito Santo – UFES, Vitória, ES Brazil

**Keywords:** Comet assay, Micronucleus test, Splenic phagocytosis, Chemotherapy, Resorcinolic lipid, Cyclophosphamide

## Abstract

**Background:**

A large number of studies are attempting to identify alternative products from natural sources or synthesized compounds that effectively interact with cancer cells without causing adverse effects on healthy cells. Resorcinolic lipids are a class of bioactive compounds that possess anticancer activity and are able to interact with the lipid bilayer. Therefore, the objective of this study was to synthesize a novel resorcinolic lipid and test its biological proprieties.

**Methods:**

We aimed to synthesize a novel resorcinolic lipid belonging to the class of cytosporones, AMS049 (3-Heptyl-4,6-dihydroxy-3*H*-isobenzofuran-1-one) and to evaluate the toxicity of two concentrations of this lipid (7.5 and 10 mg/kg) by determining its genotoxic, mutagenic, immunomodulatory, and apoptotic effects, as well as any biochemical and histopathological alterations in mice treated with cyclophosphamide. The results were analyzed by ANOVA followed by the Tukey test A . level of significance of p < 0.05 was adopted.

**Results:**

The new cytosporone AMS049 was synthesized in only three steps and in satisfactory yields. The results indicate that the compound is neither genotoxic nor mutagenic and does not alter biochemical parameters. The histological alterations observed in the liver and kidneys did not compromise the function of these organs. Histology of the spleen suggested immunomodulation, although no changes were observed in splenic phagocytosis or differential blood cell count. The results also show that AMS049 potentiates the mutagenic effect of the chemotherapy drug cyclophosphamide and that the combination induces apoptosis.

**Conclusion:**

These facts indicate a potential therapeutic application of this novel cytosporone as an important chemotherapeutic adjuvant.

**Electronic supplementary material:**

The online version of this article (doi:10.1186/s12885-015-1532-2) contains supplementary material, which is available to authorized users.

## Background

A major difficulty in developing new drugs for the treatment of cancer is the limited specificity of these compounds, which often also affect non-tumor cells. Therefore, a large number of studies are attempting to identify alternative products from natural sources or synthesized compounds that effectively interact with cancer cells without causing adverse effects on healthy cells [1] or that potentiate the effects of chemotherapy drugs and reduce their side effects [2].

To evaluate new compounds, mutagenesis studies that involve predictive testing for cancer combined with immunomodulation and apoptosis assays are a good alternative to outline and guide strategies for the prevention and/or treatment of cancer [2, 3].

Phenolic lipids (especially resorcinolic lipids) are indicated for the prevention and/or treatment of cancer. The amphiphilic property of these compounds, which is attributed to the presence of non-isoprenoid side chains bound to the hydroxybenzene ring, permits their interaction with the lipid bilayer [4–6], the formation of liposomes [7], protection against oxidative stress [8] and the inhibition of bacterial [9–11] and tumor cell growth [12, 13]. Particularly interesting are phenolic lipids of the cytosporone class, which present allelopathic activity [14] and can interact with the orphan nuclear receptor Nur77, triggering apoptosis [15]. This observation is supported by a study from our research group showing that the synthetic resorcinolic lipid 3-heptyl-3,4,6-trimethoxy-3*H*-isobenzofuran-1-one (AMS35AA) has anti-genotoxic and immunostimulatory activities, potentiates the mutagenic effect of the chemotherapy drug cyclophosphamide and increases the rate of apoptosis induced by this drug [2].

Continuing our studies on the synthesis and characterization of new phenolic lipids and the evaluation of their biological activities, the objectives of the present study were to design and synthesize a novel cytosporone and to evaluate its toxicity by determining its genotoxic, mutagenic, immunomodulatory and apoptotic effects, as well as any biochemical and histopathological alterations in Swiss mice treated with cyclophosphamide.

## Methods

### Synthesis

*Pro analysis* (P.A.) grade solvents and reagents were purchased from Acros® and Merck® and were purified, if necessary, following routine procedures.

Thin-layer chromatography (TLC) was performed on silica gel 60 F_254_ (Merck®), and the chromatograms were developed in a solution of vanillin in sulfuric acid, followed by charring.

Compounds were purified by column chromatography using silica gel (230 - 400 mesh ASTM) as the stationary phase under pressure or by high-performance liquid chromatography (HPLC) on a Shimadzu® LC 6 AD chromatograph using a Shim-pack PREP-ODS (H) (260 × 20 mm) column and UV-visible diode detector. The solvents were evaporated in a Fisaton® 802D rotary evaporator.

The samples were weighed on an analytical balance (Scientech®) with a precision ± 0.0001 g. The melting point of the compounds was determined using a Quimis® 0340S23 melting point apparatus.

The nuclear magnetic resonance (NMR) spectra were recorded on a Bruker Avance DPX-300 spectrometer at frequencies of 300 and 75 MHz for the acquisition of the ^1^H and ^13^C signals, respectively. The internal reference standard was adjusted from the TMS signal and from residual signals of the solvents present in the deuterated solvent used to solubilize the sample (CDCl_3_ or acetone-*d*_*6*_).

Mass spectra were obtained using an electron impact mass spectrometer (70 eV) coupled to a gas chromatograph (Shimadzu®, CGMS QP2010 Plus). An RTx®-Wax column from Restek® (crossbond, carbowax, polyethylene glycol; 30 m, 0.25 mm ID and 0.25 μm df) was used. Helium gas was used as the mobile phase. The ultra-high resolution and accuracy mass spectrometry (electrospray ionization Fourier transform ion cyclotron resonance mass spectrometry, ESI-FT-ICR MS, model 9.4 T Solarix, Bruker Daltonics, Bremen, Germany) was used to define the elemental composition (C_c_H_h_N_n_O_o_S_s_), DBE^1^ and *m/z* values over a mass range of *m/z* 200-2000. A resolving power, m/Δm_50%_ = 460 000, in which Δm_50%_ is the full peak width at half-maximum peak height, of *m/z ≅* 400 and a mass accuracy of < 2 ppm provided the unambiguous molecular formula assignments for singly charged molecular ions.

Phthalide 1 was prepared according to the procedures described by Navarro et al. [2], in 93 % yield.

#### Synthesis of compound 2 (3-heptyl-4,6-dimethoxy-3H-isobenzofuran-1-one)

Compound 1 (0.259 mmol, 76 mg) was solubilized in anhydrous ethanol (6 mL) and transferred to a sealed tube. NaBH_4_ (1.293 mmol) was then added to the solution. The system was completely sealed and stirred in an oil bath at 120 °C for 12 hours. The tube was then cooled to room temperature, and drops of glacial acetic acid were added until the solution became transparent. The solvent was distilled off, and the product was solubilized in CH_2_Cl_2_, poured into a separatory funnel and washed with NaCl dilluted solution twice. The organic phase was then separated, dried over MgSO_4_ and filtered, and the solvent was distilled under reduced pressure. The product was purified by chromatography on a silica gel column using a mixture of hexane:ethyl acetate (10:1, v/v) as the eluent, giving a white solid (p.f.: 80 - 81 °C) with 76 % yield.

^1^H NMR (CDCl_3_, 300 MHz). δ (ppm): 0.84 (*t*, *J* = 6.9 Hz, 3H); 1.22 (*m*, 10H); 1.61 (*m*, 1H); 2.16 (*m*, 1H); 3.82 (*s*, 3H); 3.83 (*s*, 3H); 5.41 (*dd*, *J*_1_ = 8.0 Hz and *J*_2_ = 2.9 Hz, 1H); 6.63 (*d*, *J* = 1.9 Hz, 1H); 6.87 (*d*, *J* = 1.9 Hz, 1H). ^13^C NMR (CDCl_3_, 75 MHz). δ (ppm): 14.1 (CH_3_) 22.6 (CH_2_); 24.7 (CH_2_); 29.1 (CH_2_); 29.3 (CH_2_); 31.7 (CH_2_); 32.9 (CH_2_); 55.6 (CH_3_); 55.9 (CH_3_); 80.8 (CH); 98.4 (CH); 104.8 (CH); 128.6 (C); 131.3 (C); 154.9 (C); 162.3 (C); 170.9 (C). IR (KBr tablet). ν_max/_cm^−1^: 775, 860, 953, 1038, 1111, 1157, 1335, 1354, 1428, 1466, 1504, 1763, 2361, 2847, 2920, 3005. IE-MS fragments: 292 (M^+.^), 281, 253, 207, 193 (base peak), 165, 150, 135, 107, 77, 55.

#### Synthesis of compound 3 - cytosporone AMS049 (3-heptyl-4,6-dihydroxy-3H-isobenzofuran-1-one)

Compound 2 (0.448 mmol, 130 mg) was solubilized in a solution of 5 mL BBr_3_ in CH_2_Cl_2_ (1 M) into a two-neck flask, which was completely closed and kept under magnetic agitation. After 2 hours, drops of a diluted solution of HCl (5 mL) were added. The mixture was transferred to a separatory funnel, and the organic phase (CH_2_Cl_2_) was separated. The product was extracted from the aqueous phase with CH_2_Cl_2_ (2 × 10 mL). The organic phases were combined and washed with a diluted solution of Na_2_CO_3_ (1 × 5 mL). The resulting organic phase was dried over MgSO_4_, filtered and concentrated. The crude organic product was purified by chromatography on a silica column (eluent 1/1, hexane:AcOEt, v/v), giving a white solid (83 % yield). ^1^H NMR (acetone-*d*_*6*_, 300 MHz). δ (ppm): 0.84 (t, J = 6.3 Hz, 3H); 1.25 - 1.31 (m, 10H); 1.66 - 1.73 (m, 1H); 2.20 - 2,24 (m, 1H); 5.43 - 5.46 (dd, *J*_*1*_ = 7.8 Hz, *J*_*2*_ = 3.0 Hz, 1H); 6.68 (d, *J* = 1.8 Hz, 1H); 6.72 (d, *J* = 1.8 Hz, 1H); 8.96 (sl, 1H); 9.28 (sl, 1H). ^13^C NMR (acetone-*d*_*6*_, 75 MHz). δ (ppm): 13.7 (CH3); 22.6 (CH2); 24.8 (CH2); 29.2 (CH2); 29.4 (CH2); 31.8 (CH2); 33.1 (CH2); 80.3 (CH); 101.8 (CH); 108.3 (CH); 128.2 (C); 129.1 (C); 153.3 (C); 160.1 (C); 170.4 (C). IR (KBr tablet). ν_max/_cm^−1^: 775, 868, 933, 1007, 1103, 1308, 1354, 1473, 1520, 1624, 1720, 2854, 2924, 2955, 3198. IE-MS fragments: 264 (M^+.^), 253, 207, 191, 165 (base peak), 137, 133, 109, 96, 73, 57, 45.

### Chemical agents, animals, and experimental design

Cyclophosphamide (Fosfaseron®, Laboratórios Ítaca, REG. M.S. No. 1.2603.0056.002-1; Batch 063020, Brazil), an indirectly acting alkylating agent, was used as a positive control for the induction of DNA damage. The DNA damage-inducing agent was prepared in saline, pH 7.4, and administered intraperitoneally (*ip*) as a single dose at a final concentration of 100 mg/kg body weight (bw).

The cytosporone AMS049 was first diluted in ethanol (1 %) and then in Milli-Q water (1 % final ethanol concentration) and administered at doses of 7.5 and 10 mg/kg bw (*ip*) [16].

Sixty-five sexually mature, male Swiss mice (*Mus musculus*) obtained from the Central Vivarium of the Center for Biological and Health Sciences, Federal University of Mato Grosso do Sul (Centro de Ciências Biológicas e da Saúde, Universidade Federal de Mato Grosso do Sul - CCBS/UFMS) were subdivided into two batches of 6 and 7 experimental groups (n = 5 animals). The first batch was used for the peripheral blood micronucleus test, splenic phagocytosis assay, differential blood cell count, and histopathological analysis. The second batch was used for the comet assay, biochemical analysis, and apoptosis assay. The animals were housed in propylene boxes covered with sawdust and were fed commercial chow (Nuvital®) and filtered water *ad libitum*. The animals were maintained at a controlled temperature (22 ± 2 °C) and humidity (55 ± 10 %) on a ventilated cage rack (Alesco®) with a photoperiod of 12 hours light/12 hours dark. The experiment was conducted in accordance with the guidelines of the Ethics Committee on the Use of Animals of Federal University of Mato Grosso do Sul (Protocol Nos. 399/2012 and 523/2013) and the Universal Declaration of Animal Rights.

The following experimental groups were established:

Group 1 – Negative Control: The animals simultaneously received one dose of saline (cyclophosphamide vehicle, *ip*) and one dose of saline plus 1 % ethanol (AMS049 vehicle, *ip*), each in a volume of 0.1 mL/10 g bw.

Group 2 – Positive Control – Cyclophosphamide: The animals received one dose of cyclophosphamide (100 mg/kg bw, *ip*) and one dose of saline plus 1 % ethanol (AMS049 vehicle, *ip*) in a volume of 0.1 mL/10 g bw.

Groups 3 and 4 – AMS049: The animals received one dose of saline (cyclophosphamide vehicle, 0.1 mL/10 g bw, *ip*) and one dose of AMS049 at the concentration of 7.5 (group 3) or 10 (group 4) mg/kg bw, *ip*.

Groups 5 and 6 – Combined: The animals simultaneously received one dose of AMS049 (at concentrations of 7.5 (group 5) or 10 (group 6) mg/kg bw, *ip*) and one dose of cyclophosphamide (100 mg/kg bw, *ip*).

Group 7 – Naive: The animals did not receive any type of treatment. This group was used only in the second batch of animals used for biochemical evaluation.

For animals in the first batch, peripheral blood samples (20 μL) were collected for the micronucleus test at 24 (T1), 48 (T2), and 72 hours (T3) after administration of the compounds. At T3, 20 μL peripheral blood was also collected for differential blood cell analysis. Seventy-two hours after application of the test compounds, the animals were sacrificed by cervical dislocation for organ collection. The organs were weighed and used for histopathology and evaluation of splenic phagocytosis. For animals in the second batch, 20 μL peripheral blood was collected 24 hours (T1) after administration of the test compounds for the comet assay. After 72 hours, the animals were anesthetized by intramuscular injection of 50 mg/kg ketamine and 10 mg/kg xylazine, and blood was collected for the biochemical assays by complete exsanguination through the orbital plexus [17]. The animals were then euthanized by cervical dislocation, and organs were collected for the apoptosis assay.

### Biological assays

#### Comet assay

The comet assay was performed as described by Singh et al. [18]. For this purpose, 20 μL peripheral blood and 120 μL low-melting point (LMP) agarose (0.5 %) were homogenized. This solution was transferred to slides previously coated with normal agarose (5 %), and the biological material was covered with a glass coverslip. The slides were cooled at 4 °C for 20 minutes. Next, the coverslips were removed, and the slides were immersed in freshly prepared lysis solution (89 mL stock lysis solution – 2.5 M NaCl; 100 mM EDTA; 10 mM Tris, pH 10.0, adjusted with solid NaOH; 1.0 mL Triton X-100; and 10.0 mL DMSO). Lysis was performed for 1 hour at 4 °C protected from light. The slides were then transferred to an electrophoresis chamber containing buffer with a pH > 13.0 (300 mM NaOH and 1 mM EDTA, prepared from a stock solution of 10 N NaOH and 200 mM EDTA, pH 10.0) for 20 minutes at 4 °C for DNA denaturation. Electrophoresis was performed at 25 V and 300 mA (1.25 V/cm) for 20 minutes. After electrophoresis, the slides were neutralized in 0.4 M Tris-HCl (pH 7.5) for three cycles of 5 minutes each, air dried and fixed in absolute ethyl alcohol for 10 minutes. The material was stained with 100 μL ethidium bromide (20 × 10^−3^ mg/mL) and analyzed under an epifluorescence microscope (Bioval®) at 40× magnification using a 420 - 490 nm excitation filter and a 520 nm barrier filter. As described by Kobayashi et al. [19], 100 cells per animal were analyzed, and comets were classified as follows: (class 0) undamaged cells with no tail, (class 1) cells with a tail smaller than the diameter of the nucleus, (class 2) cells with a tail length one to two times the diameter of the nucleus and (class 3) cells with a tail length greater than two times the diameter of the nucleus. Apoptotic cells containing a completely fragmented nucleus were not counted. The total score was calculated from the sum of the values resulting from multiplication of the total number of cells observed in each damage class to which they belonged by the value of the class.

#### Peripheral blood micronucleus test

The peripheral blood micronucleus test was performed according to Hayashi et al. [20] and modified by Oliveira et al. [21].

#### Apoptosis assay

One hundred microliters of a solution of macerated liver or kidney was used for smear preparation. The slide was fixed in Carnoy’s fixative for 5 minutes and subjected to a decreasing ethanol series (95 % - 25 %). Next, the slide was washed with McIlvaine buffer for 5 minutes, stained with acridine orange (0.01 %, 5 minutes) and again washed with buffer. Apoptotic cells were identified by analyzing the DNA fragmentation patterns according to Rovozzo and Burke [22] and Mauro et al. [23].

#### Splenic phagocytosis assay

The spleen was macerated in saline. One hundred microliters of the cell suspension was transferred to a slide previously stained with 20 μL acridine orange (1.0 mg/mL) and coverslipped. The slides were stored in a freezer until analysis. The slides were analyzed under a fluorescence microscope (Bioval®, Model L 2000A) at 400× magnification using an excitation filter of 420 - 490 nm and a barrier filter of 520 nm [24]. A total of 200 cells per animal were analyzed. The absence or presence of phagocytosis was defined based on the description of Hayashi et al. [20].

#### Differential blood cell count

Twenty microliters of peripheral blood was used for smear preparation on a histology slide. The slides were air dried, stained with 10 % Giemsa for 10 minutes, and examined by bright-field microscopy at 1000× magnification. A total of 100 cells/animal were analyzed and differentiated into lymphocytes, neutrophils, monocytes, eosinophils and basophils.

#### Biochemical assays

After the collection and sedimentation of the blood samples in a refrigerator (4 °C), the serum was separated and stored in a freezer (-10 °C) until analysis. The following biochemical parameters were analyzed in an automated Cobas 600 analyzer (Roche Diagnostics®) according to manufacturer specifications: aspartate aminotransferase (AST); alanine aminotransferase (ALT); urea; creatinine; and Na^+^, K^+^, Ca^2+^ and Mg^2+^ ions.

#### Histopathological analysis

The liver, spleen, and kidneys were cut, and the fragments were fixed in 10 % neutral buffered formalin and submitted to routine processing for histological analysis. Briefly, the fixed tissue fragments were dehydrated, cleared, and embedded in paraffin. The samples were then cut into 6 μm-thick sections and stained with hematoxylin-eosin for histopathological analysis.

#### Calculation of the percentage of damage reduction (%DR) and damage increase (%DI)

Manoharan and Beneriee [25] and Waters [26] proposed a calculation of the percentage of damage reduction to evaluate the chemopreventive capacity of a substance when it is combined with a known mutagenic agent. In the present study, the test compound showed anti-genotoxic but not antimutagenic activity. In the latter case, an increase in the frequency of DNA damage was observed. Therefore, in the present study, the percentage of damage reduction and the percentage of damage increase were calculated using the same formula.

$$ \mathrm{D}\mathrm{R}\%\;\mathrm{or}\;\mathrm{D}\mathrm{I}\%=\frac{Mean\  of\  positive\  control\hbox{--} Mean\  of\  combination\  group}{Mean\  of positive\  control\hbox{--} Mean\  of negative\  control}\times 100 $$

#### Statistical analysis

The results are expressed as means ± standard errors (SE) and were analyzed by ANOVA followed by the Tukey test using the GraphPad Prism software (version 3.02; Graph-Pad Software, Inc., San Diego, CA, USA). A level of significance of p < 0.05 was adopted.

## Results

### Planning and synthesis of the novel cytosporone

**Fig. 1:**
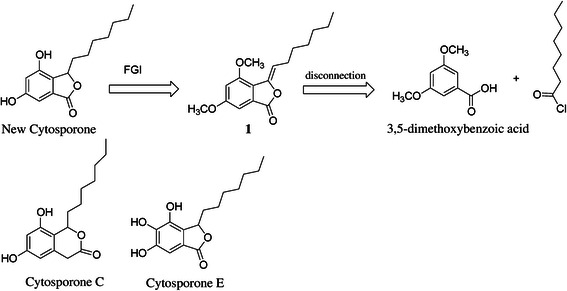
Retrosynthetic analysis of the novel cytosporone and structures of natural cytosporones C and E. FGI – Functional Group Interconversion

Figure 1 illustrates the retrosynthetic analysis of a new cytosporone. The target compound is achieved by the key-intermediate (phthalide 1) after hydrogenation followed by demethylation reactions. Compound 1 was planned to be obtained by the showed disconnection, leading to 3,5-dimethoxybenzoic acid and octanoyl chloride as simpler starting materials.

The designed synthetic cytosporone is structurally similar to cytosporones C and B isolated by Brady [27], especially in the presence of a lactone ring fused to the aromatic ring. Cytosporone C is a δ-lactone resorcinol, whereas the designed cytosporone and cytosporone E are γ-lactones. However, the latter is hydroxylated at C-4.

**Fig. 2:**
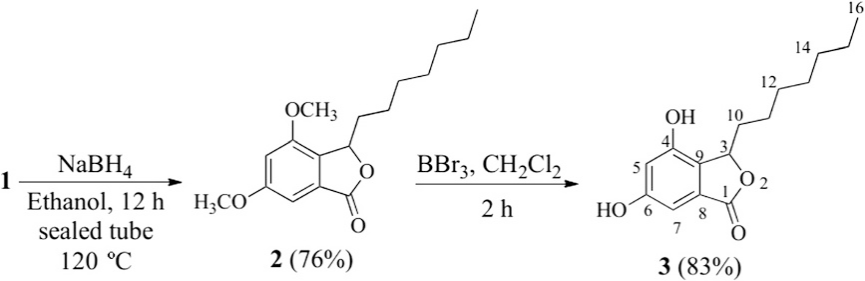
Synthesis of AMS049

The phthalide 1 was obtained as a product of the Friedel-Crafts acylation of 3,5-dimethoxybenzoic acid [2], which was treated with NaBH_4_ in ethanol at 120 °C in a sealed tube. The workup was made after 12 hours, and the compound was purified on a silica gel column and analyzed by TLC and NMR spectroscopy, which revealed the formation of only one product of interest (compound 2) in 76 % yield after purification on a silica gel column. Compound 2 was then treated with BBr_3_ in dichloromethane at room temperature for 2 hours in order to remove the methyl groups. This treatment provided the new cytosporone (compound 3), termed AMS049, in 83 % yield (Fig. 2).

Compounds 2 and 3 were mainly identified based on ^1^H and ^13^C NMR spectral data.

The ^1^H NMR spectrum of compound 2 showed a double doublet at 5.41 ppm, an important signal associated with the hydrogen of the stereogenic center formed during reduction of the double bound of C-3. This reduction converts the second carbon of the lipophilic chain, previously an *sp*^*2*^ hybridized carbon, into a diastereotopic CH_2_ (C-10), which produces two multiplets (1.58 - 1.65 and 2.15 - 2.29 ppm). In addition to the disappearance of the signal for olefinic hydrogen above 6.00 ppm, the other signals were compatible with the structure of compound 2. The ^13^C NMR spectrum, showed a signal at 170.9 ppm that was assigned to the carbon of the lactone carbonyl (C-1). The signal of the stereogenic carbon (C-3) appeared at 80.8 ppm. These signals are in accordande with heteronuclear single quantum coherence spectroscopy (HSQC) and heteronuclear multiple-bond correlation spectroscopy (HMBC) experiments, corroborating to characterize the structure of the product.

FTIR spectrum of compound 2 showed important bands, such as that of the above alkyl C-H stretch slightly below 3,000 cm^−1^; stretching of the C-O bond of the carbonyl ester at 1,763 cm^−1^; and C-H bond deformations in the aromatic ring at 953, 860 and 775 cm^−1^, a *meta*-substitution pattern.

The ^1^H NMR spectrum of cytosporone 3 did not show the signals corresponding to aromatic methoxyl groups above 3.50 ppm, but the presence of two large singlets at 8.96 and 9.28 ppm corresponding to phenolic hydrogens. The other signals were the same as those observed in the ^1^H NMR spectrum of precursor 2. Disappearance of the signals corresponding to the carbons of the methoxyl groups was noted in the ^13^C NMR spectrum, demonstrating the complete deprotection of the product. Tables 1 and 2 show the ^1^H and ^13^C NMR spectral data for the non-isoprenoid lipids 2 and 3, respectively (^1^H and ^13^C NMR spectra of compounds 2 and 3 are available in Additional files 1, 2, 3, 4 and 5).

**Table 1:** NMR data related to compounds 2 and 3 (^1^H at 300 MHz, CDCl3)

Hydrogen	Compound	
	2^a^ δ(ppm) (m/*J* Hz)	3^b^ δ(ppm) (multiplicity/*J* Hz)
3	5,41 (*dd*/*J*_1_ = 8,0/*J*_2_ = 2,9)	5,45 (*dd*/*J*_*1*_ = 7,8/*J*_*2*_ = 3,0)
5	6,63 *(d*/1,9)	6,68 (*d*/1,8)
7	6,87 *(d*/1,9)	6,72 (*d*/1,8)
10	2,16 e 1,61 *(m)*	2,22 e 1,70 (*m*)
11	1,22 *(m)*	1,25-1,31 (*m*)
12	1,22 *(m)*	1,25-1,31 (*m*)
13	1,22 *(m)*	1,25-1,31 (*m*)
14	1,22 *(m)*	1,25-1,31 (*m*)
15	1,28	1,25-1,31 (*m)*
16	0,83 (*t/J* = 6,9)	0,84 (*t/J* = 6,3)
C-4-OCH_3_	3,82 (*s*)	-
C-6-OCH_3_	3,83 (*s*)	-
Ph-OH	-	9,28 e 8,96 (*sl*)

^a^Solubilized in CDCl_3_. ^b^Solubilized in Acetone-*d*_6_

**Table 2:** ^13^C NMR data of the compounds 2 and 3 (75 MHz, CDCl_3_)

Carbon	Compound	
	2^a^	3^b^
1	170,9	170,4
3	80,8	80,3
4	154,9	153,3
5	104,8	108,3
6	162,3	160,1
7	98,4	101,8
8	128,6	128,2
9	131,3	129,1
10	31,7	31,8
11	24,7	24,8
12	29,3	29,4
13	29,1	29,2
14	32,9	33,1
15	22,6	22,6
16	14,1	13,7
C-4-OCH_3_	55,9	-
C-6-OCH_3_	55,6	-

^a^Solubilized in CDCl_3_. ^b^Solubilized in Acetone-*d*_*6*_

**Fig. 3:**
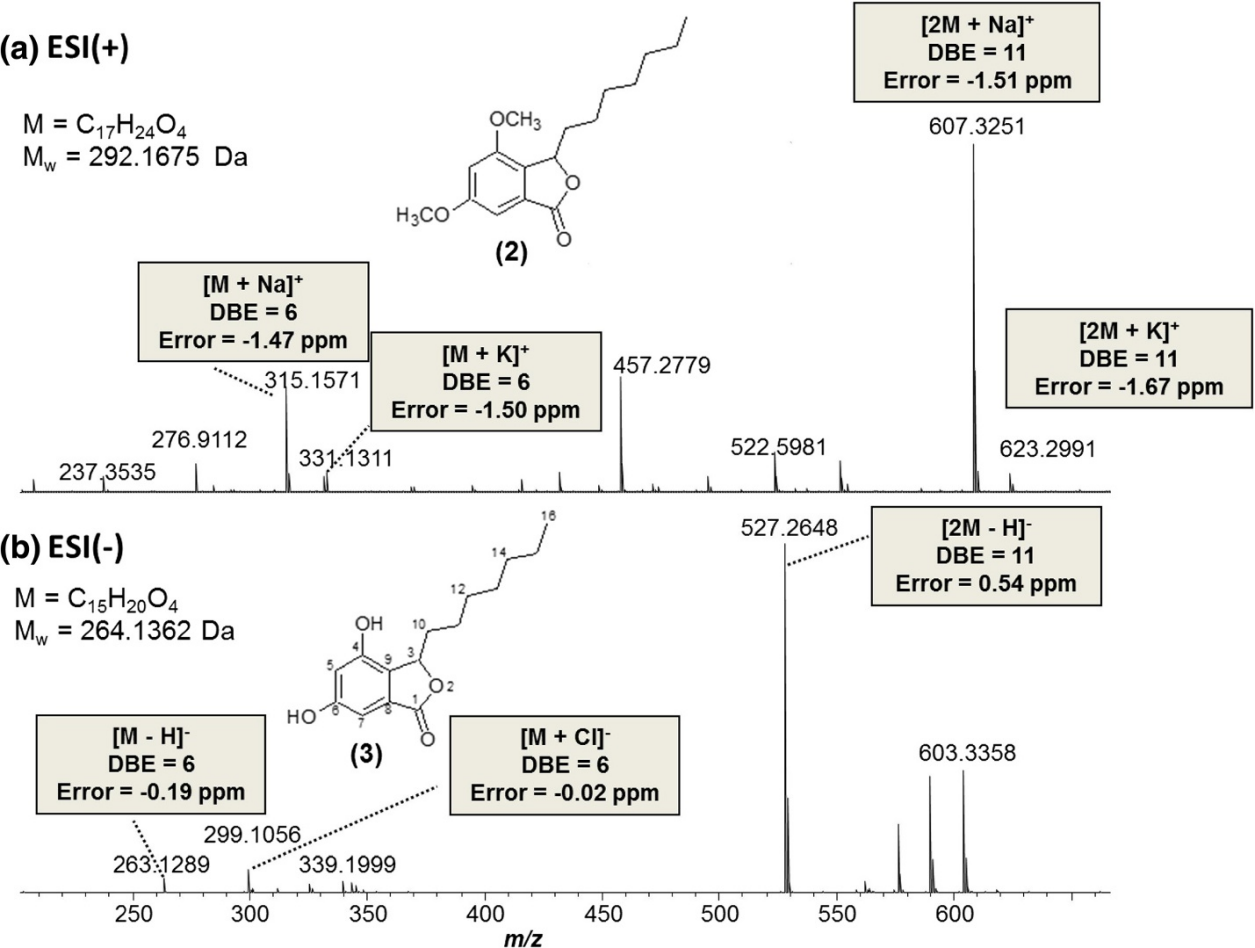
(**a**) ESI(+) and (**b**) ESI(-)FT-ICR mass spectra for compounds 2 and 3, respectively

Figure 3a-b show ESI-FT-ICR mass spectra for compound 2 and 3, respectively. For compound 2, Fig. 3a, ESI(+)-FT-ICR MS provides an unambiguous molecular formula (M) of C_17_H_24_O_4_, where [M + Na]^+^, [M + K]^+^, [2 M + Na]^+^ and [2 M + K]^+^ ions with *m/z* of 315.1571, 331.1311, 607.3251 and 623.2991 are identified, respectively. All present an accuracy mass < 2 ppm. The DBE = 6 for the [M + Na]^+^ and [M + K]^+^ ions allow indicating the presence of an aromatic (DBE = 4) and one furan (DBE = 1) rings, and one ketone group (DBE =1).

For compound 3, Fig. 3b, ESI(-)-FT-ICR MS provides M of C_15_H_20_O_4_, where the [M - H]^−^, [M + Cl]^−^, and [2 M - H]^−^ ions with *m/z* of 263.1289 (DBE = 6), 299.1056 (DBE =6) and 527.2648 (DBE = 11) are identified, respectively. All have an accuracy mass < 1 ppm.

### Biological assays

**Fig. 4:**
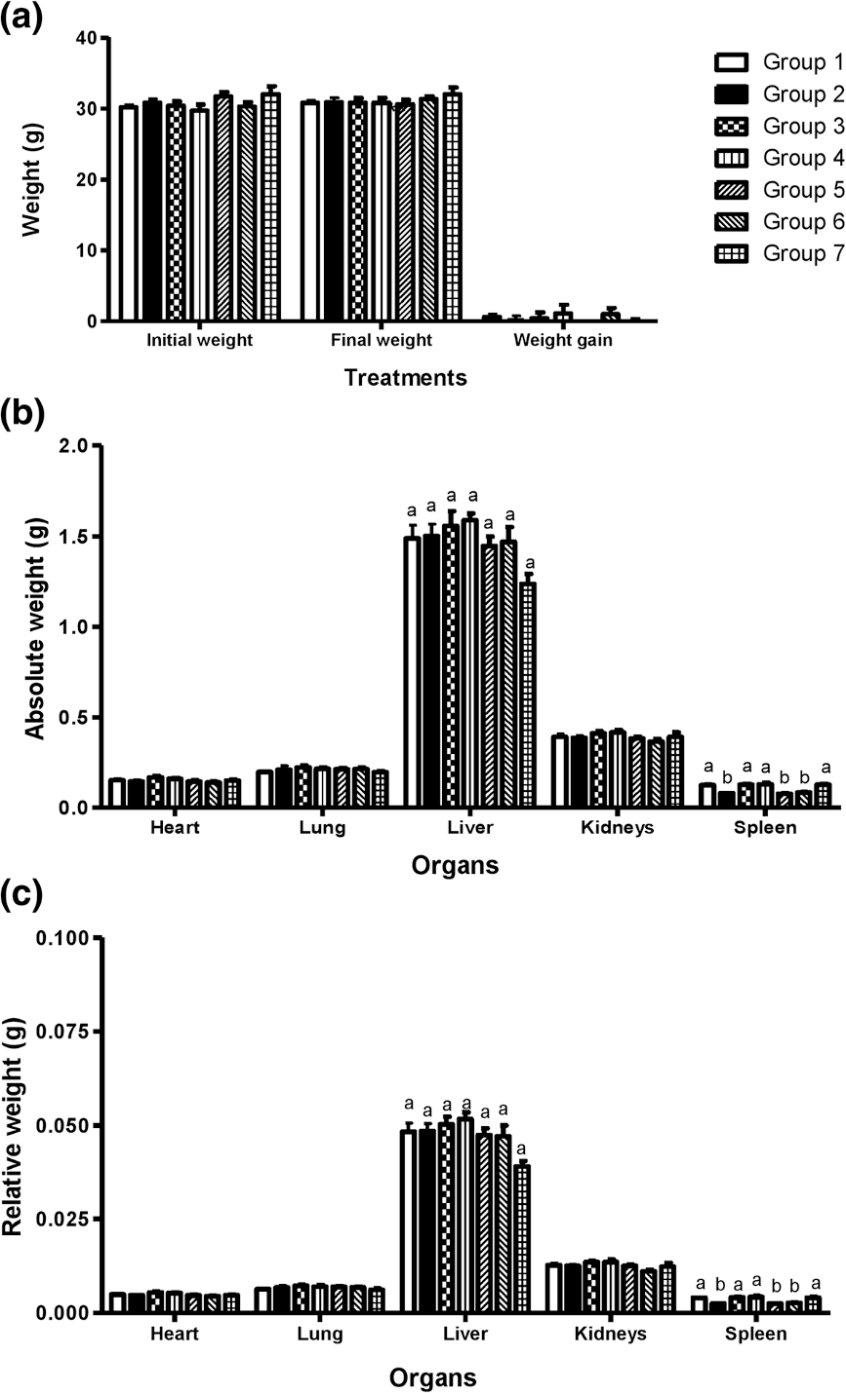
Data of mice’ weights represented as graphic. **a** Initial, final and weight gain. **b** Absolute weight of the organs. **c** Relative weight of the organs. Different letters indicate statistically significant differences (p < 0.05; ANOVA/Tukey test)

No significant differences were observed in the initial and final weight of the animals; weight gain; or absolute and relative weight of the heart, liver, lungs and kidneys (p > 0.05). Absolute and relative spleen weight was lower in the groups treated with cyclophosphamide alone or in combination with AMS049 (p < 0.05) (Fig. 4).

The cytosporone tested (AMS049) was not genotoxic and exhibited discrete anti-genotoxic activity when administered in combination with cyclophosphamide (p > 0.05). The %DRs were 33.02 and 33.33 % for the doses of 7.5 and 10 mg/kg, respectively (Table 3).

**Table 3:** Results related to anti-genotoxicity tests of AMS049 through the comet assay in peripheral blood cells

Experimental groups	Damaged cells	Damage classes				Score	%DR
		0	1	2	3		
Group 1	22.80 ± 5.04^a^	77.40 ± 5.07	20.00 ± 4.86	2.80 ± 0.80	0.00 ± 0.00	25.60 ± 5.34^a,b^	-
Group 2	86.40 ± 3.15^c^	13.20 ± 3.38	64.00 ± 2.02	17.20 ± 1.88	5.20 ± 1.39	114.00 ± 7.45^c^	-
Group 3	30.80 ± 4.39^a,b^	69.20 ± 4.40	28.40 ± 3.96	2.40 ± 0.75	0.00 ± 0.00	33.20 ± 4.91^a,b^	-
Group 4	48.80 ± 8.89^b^	51.20 ± 8.90	36.00 ± 4.61	12.40 ± 4.57	0.40 ± 0.40	62.00 ± 13.98^b^	-
Group 5	65.40 ± 3.01^b,c^	34.60 ± 3.01	47.20 ± 2.59	13.80 ± 1.98	4.40 ± 1.57	88.00 ± 5.26^b,c^	33.02
Group 6	65.20 ± 5.81^b,c^	34.80 ± 5.81	50.20 ± 3.95	11.20 ± 3.93	3.80 ± 2.33	84.00 ± 13.52^b.c^	33.33
Group 7	NA	NA	NA	NA	NA	NA	NA

SE: Standard error

NA: Not analysed

%DR: Percentage of damage reduction

Different letters indicate statistically significant differences (p < 0.05; ANOVA/Tukey)

AMS049 exhibited no mutagenic activity at any dose or time of evaluation. When compound 3 was administered in combination with cyclophosphamide, increases (p < 0.05) in the percentage of mutagenic damage by 38.88 and 35.35 % were observed at T1 with doses of 7.5 and 10 mg/kg, respectively. In contrast, the frequency of micronuclei could not be determined at T2 and T3 because of the small number of analyzable red blood cells (Table 4).

**Table 4:** Total frequency and mean ± SE of the micronucleus assay in peripheral blood cells

Experimental groups	Mean ± SE			%DI
	24 h (T1)	48 h (T2)	72 h (T3)	
Group 1	8.80 ± 0.58ª	10.40 ± 2.31ª	13.20 ± 1.74ª	-
Group 2	48.40 ± 1.21^b^	38.80 ± 2.96^b^	35.74 ± 1.79^b^	-
Group 3	12.40 ± 2.29ª	7.40 ± 0.81ª	12.40 ± 1.50ª	-
Group 4	9.20 ± 1.11ª	7.00 ± 0.89ª	14.00 ± 0.95ª	-
Group 5	63.80 ± 6.89^c^	NA	NA	38.81
Group 6	62.40 ± 1.36^c^	NA	NA	35.35
Group 7	NA	NA	NA	NA

SE: Standard error

NA: Not analysed

%DI: Percentage of damage increase

Different letters indicate statistically significant differences (p < 0.05; ANOVA/Tukey)

The administration of AMS049 resulted in 15.28- and 18.12-fold increases in the frequency of hepatic apoptosis at doses of 7.5 and 10 mg/kg, respectively, when compared with the controls (p < 0.05). In the kidneys, this frequency increased 15.20-fold and 15.51-fold at the same doses. When cyclophosphamide was administered alone, the frequency of apoptosis increased 21.08-fold in the liver and 15.14-fold in the kidneys compared with the controls (p < 0.05). In contrast, the combined administration of cyclophosphamide and AMS049 at doses of 7.5 and 10 mg/kg resulted in 1.68- and 1.74-fold increases in the frequency of apoptosis in the liver and 2.15- and 2.37-fold increases in the kidneys, respectively, when compared with the cyclophosphamide group (p < 0.05) (Table 5).

**Table 5:** Apoptosis evaluation on mice’ kidneys and liver

Experimental groups	Liver		Kidneys	
	Number of apoptotic cells	Mean ± SE	Number of apoptotic cells	Mean ± SE
Group 1	64	12.80 ± 2.40^a^	69	13.80 ± 1.88ª
Group 2	1349	269.80 ± 22.63^b^	1045	209.00 ± 42.42^b^
Group 3	978	195.60 ± 4.69^b^	1049	209.80 ± 8.63^b^
Group 4	1160	232.00 ± 20.38^b^	1070	214.00 ± 36.08^b^
Group 5	2272	454.40 ± 24.35^c^	2243	448.60 ± 24.92^c^
Group 6	2345	469.00 ± 38.45^c^	2480	496.00 ± 24.03^c^
Group 7	NA	NA	NA	NA

SE: Standard error

NA: Not analyzed

Different letters indicate statistically significant differences (p < 0.05; ANOVA/Tukey)

The administration of cyclophosphamide alone or in combination with AMS049 reduced the number of cells in the spleen (p < 0.05), demonstrating that phagocytosis had occurred. In contrast, no changes were observed when compound 3 was administered alone (Table 6).

**Table 6:** Results related to splenic phagocytosis

Experimental Groups	Number of analyzed cells	Total of cells without phagocytosis evidence			Total of cells with phagocytosis evidence		
		Absolute values	Mean ± SE	Percentage (%)	Absolute values	Mean ± SE	Percentage (%)
Group 1	1000	241	48.20 ± 4.95ª	24.10	759	151,.80 ± 4.95ª	75.90
Group 2	1000	483	96.60 ± 1.85^b^	48.30	519	103.80 ± 1.88^b^	51.90
Group 3	1000	251	50.20 ± 4.15ª	25.10	749	149.80 ± 4.15ª	74.90
Group 4	1000	200	40.00 ± 4.78ª	20.00	800	160.00 ± 4.78ª	80.00
Group 5	1000	464	92.80 ± 2.01^b^	46.40	536	107.20 ± 2.01^b^	53.60
Group 6	1000	471	94.20 ± 2.58^b^	47.10	529	105.80 ± 2.57^b^	52.90
Group 7	NA	NA	NA	NA	NA	NA	NA

SE: Standard error

NA: Not analyzed

Different letters indicate statistically significant differences (p < 0.05; ANOVA/Tukey)

The differential blood cell count indicated no differences in the numbers of lymphocytes or basophils (p > 0.05), which were within the reference range. In contrast, eosinophil counts were higher than the reference range, although no significant differences were observed between groups. Despite the lack of significant differences in the number of neutrophils (p > 0.05), groups 1, 2 and 5 presented higher numbers than the reference ranges. An increase in the number of monocytes was only observed in the group treated with 10 mg/kg AMS049 (group 4) (p < 0.05). In addition, monocyte counts higher than the reference range were observed in this group and in group 5 (Table 7).

**Table 7:** Reference values and mean ± SE of the differential blood cell count

Cell types	Reference values	Experimental groups						
		Group 1	Group 2	Group 3	Group 4	Group 5	Group 6	Group 7
Lymphocyte	55 – 95 %	46.80 ± 1.96^a,b^	40.40 ± 3,21^b^	57.60 ± 1.20^a^	54.80 ± 1.42^a^	41.60 ± 3.58^b^	57.20 ± 4.81^a^	NA
Neutrophil	10 – 40 %	48.80 ± 1.93^a,b^	55.20 ± 1,68^b^	35.40 ± 0.74^a^	37.40 ± 1.47^a^	52.60 ± 3.97^b^	37.60 ± 5.11^a^	NA
Monocyte	0.1 – 3.5 %	2.20 ± 0.73^b^	2.40 ± 1.16^a,b^	3.20 ± 0.58^a,b^	5.80 ± 0.86^a^	3.80 ± 0.73^a,b^	3.20 ± 0.58^a,b^	NA
Eosinophil	0 – 0.4 %	2.20 ± 0.58^a^	1.80 ± 0.58^a^	3.80 ± 0.58^a^	1.80 ± 0.58^a^	2.00 ± 0.54^a^	1.80 ± 0.58^a^	NA
Basophil	0 – 0.3 %	0.00 ± 0.00^a^	0.20 ± 0.20^a^	0.00 ± 0.00^a^	0.20 ± 0.20^a^	0.00 ± 0.00^a^	0.20 ± 0.20^a^	NA

SE: Standard error

Different letters indicate statistically significant differences (p < 0.05; ANOVA/Tukey)

Analysis of biochemical profiles showed no alterations in total glucose output (AST), dTDP-glucose pyrophosphorylase (ALT), urea, creatinine, sodium, potassium, calcium or magnesium (Table 8).

**Table 8:** Biochemical evaluation of mice’ peripheral blood

Experimental Groups	AST	ALT	Urea	Creatinine	Sodium	Potassium	Calcium	Magnesium
	(U/L)	(U/L)	(mg/dL)	(mg/dL)	(mEq/L)	(mmol/L)	(mg/dL)	(mg/dL)
Group 1	114.2 ± 6.53^a^	59.4 ± 8.86^a^	64.30 ± 5.88^a^	0.28 ± 0.06^a^	125.0 ± 7.59^a^	67.4 ± 10.79^a,b^	9.86 ± 0.18^a,b^	0.38 ± 0.06^a^
Group 2	158.2 ± 8.32^a,b,c^	89.2 ± 12.74^a^	58.20 ± 4.62^a^	0.26 ± 0.02^a^	134.2 ± 1.93^a^	83.6 ± 4.41^a^	9.70 ± 0.21^a,b^	0.32 ± 0.02^a^
Group 3	146.8 ± 16.08^a,b,c^	101.4 ± 16.14^a^	61.62 ± 9.59^a^	0.30 ± 0.03^a^	136.2 ± 6.52^a^	35.8 ± 11.04^b^	8.98 ± 0.27^b^	0.44 ± 0.04^a^
Group 4	163.8 ± 14.39^a,b,c^	104.4 ± 21.48^a^	61.82 ± 5.43^a^	0.32 ± 0.04^a^	144.0 ± 7.43^a^	59.1 ± 16.13^a,b^	10.10 ± 0.14^a^	0.41 ± 0.05^a^
Group 5	183.4 ± 11.57^b,c^	102.4 ± 8.72^a^	48.22 ± 2.43^a^	0.40 ± 0.04^a^	134.2 ± 2.92^a^	50.6 ± 3.57^a,b^	9.78 ± 0.29^a,b^	0.40 ± 0.03^a^
Group 6	167.6 ± 14.91^c^	98.6 ± 29.56^a^	59.70 ± 2.79^a^	0.46 ± 0.13^a^	142.2 ± 1.59^a^	42.2 ± 10.38^a,b^	10.46 ± 0.10^a^	0.42 ± 0.03^a^
Group 7	118.0 ± 6.63^a,c^	63.2 ± 7.28^a^	57.00 ± 2.90^a^	0.28 ± 0.04^a^	122.8 ± 2.35^a^	50.8 ± 5.91ª^,b^	9.86 ± 0.09^a,b^	0.38 ± 0.05ª

SE: Standard error

AST - Aspartate aminotransferase

ALT - Alanine aminotransferase

Different letters indicate statistically significant differences (p < 0.05; ANOVA/Tukey)

Histopathological analysis revealed normal liver morphology in groups 1, 2, 3, and 4, in which the lobules were filled with hepatocytes with preserved morphology, exhibiting the vascular portal triad in some areas. Cortical glomeruli with preserved architecture were observed in renal sections. The medullary renal tubules contained a lightly eosinophilic material. In contrast, a reduction in Bowman’s capsular space and congested blood vessels were observed in the kidneys of animals treated with cyclophosphamide in combination with AMS049. Histological sections of the liver showed cytoplasmic rarefaction and loss of cytoplasmic eosinophilia. Hyperplastic and reactive lymphoid follicles, forming a germinal center, were observed in splenic sections.

## Discussion

A novel synthetic cytosporone was synthesized in only three steps and with a satisfactory overall yield. The cytosporone, called AMS049 in this study, was purified and completely characterized using ^1^H and ^13^C NMR spectroscopic techniques, and mass spectrometry

Studies suggest that cancer is caused by mutational events [3] that induce the activation of proto-oncogenes and the inactivation of tumor suppressor genes [28]. However, when mutations are induced in tumor cells by chemotherapy drugs, apoptosis can also result. Therefore, the mutational event is understood to be an important part of chemotherapy.

Within this context, there is a growing interest in identifying anticancer substances [29] or compounds that potentiate the effects of commercial chemotherapy drugs [2] but without causing adverse effects on healthy cells. In this respect, an interesting approach is to design, modify and synthesize organic compounds in the laboratory to reduce these side effects, which was the objective of the present study.

Several natural or synthetic resorcinolic lipids have shown anticarcinogenic activity [1, 12, 13, 30–41], which was also suggested by this study.

Toxicogenetic tests showed that AMS049 has no genotoxic or mutagenic activity, suggesting that this compound is nontoxic and is safe for use. This result is supported by studies that demonstrated the absence of toxicity of other phenolic lipids [2, 33–35, 41].

A chemopreventive potential of cytosporone was not confirmed because the compound has no anti-genotoxic activity and is able to potentiate the mutagenic effects of the chemotherapy drug cyclophosphamide, suggesting that the combined use of these compounds is correlated with a larger number of mutational events that lead to apoptosis.

Parikka [40] and Navarro [2] reported anti-genotoxic activity for the phenolic lipids 5-*n*-alkylresorcinol (6 - 9) and 3-heptyl-3,4,6-trimethoxy-3*H*-isobenzofuran-1-one (4), respectively. If these compounds were used as chemotherapeutic adjuvants, the anti-genotoxic activity would not be adequate or desired because it may impair apoptosis.

The lipophilicity (reported as Log *P*) of these compounds and of other correlated substances is shown in Table 9. Interestingly, in the series shown in this table, the hydrophobicity of anti-genotoxic lipids 2, 4 and 6 - 10 was higher than 5, whereas that of compounds 1, 3 and cytosporone B (5) was lower than this value.

**Table 9:** Lipophilicity (Log P) related to some natural and synthetic phenolic lipids

Structure		Theoretical Log P*
	1	4.610
	2	5.016
	AMS049 (3)	4.412
	4	5.058
	Cytosporone B (5)	4.701
	6 (C:15)	8.284
	7 (C:17)	8.850
	8 (C:19)	9.209
	9 (C:21)	9.472
	10 (C:23)	9.681

*The Theoretical Log P was calculated using the MolInspiration algorithm (http://www.molinspiration.com/cgi-bin/properties)

Cytosporone B is known to have anticancer activity and acts as a natural physiological ligand for the orphan nuclear receptor Nur77 in eukaryotic cells. The results of molecular modeling were confirmed by the biological responses obtained from *in vivo* tests in mice. Agonist binding to the receptor leads to the activation of receptor-related specific genes that control apoptosis and metabolic regulation [41]. According to Liu [14], the pharmacophoric components necessary for binding to Nur77 include the hydroxybenzene ring and the hydrophobic acyl chain, whereas the key element for the activation of the biological function of Nur77 is an ester group.

In contrast to compounds 4 and 6 - 9, the synthetic cytosporone AMS049 designed and synthesized for this study exhibits part of the features mentioned above, including the presence of a phenol group as well as an ester group (a five-membered lactone). This structure does not contain the acyl group in the benzylic carbon when compared to cytosporone B (Compound 5, Table 9). However, this carbon is attached to an oxygen atom, allowing interactions with biological receptors through hydrogen bonding. Therefore, it is possible to rationalize in a preliminary manner that the anticarcinogenic activity of AMS049 is due to these structural characteristics, which also confer moderate lipophilicity (Log P 4.412), thus permitting balance between *in vivo* permeability and solubility.

The micronucleus test indicated that the simultaneous administration of AMS049 and cyclophosphamide increased the frequency of DNA damage. This action could be important for the treatment of cancer if cytosporone is used as an adjuvant because cyclophosphamide is cytotoxic to tumor cells by causing cellular injury such as DNA damage, including micronuclei, and by inducing a complex cascade of events that involve the activation of caspases and cysteine proteases [42]. A similar phenomenon was described by Navarro [2], who evaluated the synthetic resorcinolic lipid AMS35AA. However, the capacity of AMS049 to increase DNA damage is greater, as indicated by the observation that a lower dose (7.5 mg/kg) there was a DI% of 38.88 %. This number is 2.01 times higher than that reported for AMS35AA administered at a dose of 10 mg/kg.

As observed by Navarro [2], in the present study, it was not possible to quantify the frequency of micronuclei in erythrocytes of animals treated simultaneously with cyclophosphamide and AMS049 after 24 hours. This finding is likely due to the amphiphilic properties of resorcinolic lipids. According to Kozubek and Tyman [43], the stabilization of phenolic lipids and derivatives in membranes is the result of the interaction of hydroxyl groups of the aromatic ring with phospholipids via hydrogen bonds. As a consequence, phenolic lipids are rapidly and effectively incorporated into phospholipid bilayers [44–46]. This occurrence may have increased the permeability of the erythrocyte membrane to small non-electrolytes with a molecular diameter of 1.4 nm or less [47], as well as to water [5], causing lysis of the cell [48, 49]. These factors, when combined with others described in the literature such as the relationship between the hemolytic capacity of homologous resorcinolic lipids and the length and degree of unsaturation of the aliphatic side chain [47], permit the inference that AMS049 has high hemolytic activity. This explains the absence of analyzable erythrocytes in the peripheral blood micronucleus test.

The apoptosis assay demonstrated an increase in cell death when AMS049 was administered alone or in combination with cyclophosphamide. This observation is important when considering the possible combined application of this compound with a commercial chemotherapy because an increase in cell damage can trigger the death of tumor cells. In addition, chemotherapy increases the frequency of free radicals and reduces antioxidant defenses in the organism [50]. One example is the reduction of superoxide dismutase [51], which favors apoptosis of tumor cells or cells with genetic instability when compared with normal cells.

Apoptosis is characterized by cell shrinkage, chromatin condensation, and the activation of specific cysteine proteases, known as caspases [1]. Other authors have demonstrated the apoptotic activity of compounds similar to AMS049 [2, 14, 16, 41, 52–54], suggesting the induction of apoptosis by the activation of Nur77 [55, 56].

Nur77 is a unique transcription factor of the orphan nuclear receptor superfamily [56]. The protein consists of an amino-terminal transactivation domain, a DNA-binding domain, and a carboxy-terminal ligand-binding domain [57]. In cancer cells, Nur77 becomes a potent activator of cell death in response to apoptotic stimuli that induce its migration to the mitochondria. In mitochondria, Nur77 interacts with Bcl-2. This interaction induces a conformational change of Bcl-2, triggering the release of cytochrome c and, finally, apoptosis [58–60]. According to the literature, apoptosis triggered by AMS049 analogs may occur through a cross-talk between Nur77 and BRE, a death receptor-associated protein. This event is mediated specifically by repression of the transcriptional activity of BRE through recruitment of the corepressor N-CoR, in which Nur77 binds to the BRE promoter, regulating the transcription of this protein [61, 62].

Lin [58] suggested that AMS049 analogs such as cytosporone B reduce the mitochondrial membrane potential. In addition, critical events of apoptosis such as the cleavage of caspase-9 and caspase-3 are induced [14]. As discussed earlier, this is added to the activation of Nur77, which is possibly the result of the presence of an ester radical in the structure of these compounds. Thus, because AMS049 is a lactone (cyclic ester), it is possible to infer its capacity to activate Nur77, triggering apoptosis. Taken together, these observations make resorcinolic lipids, including AMS049, an interesting target for the development of new anticancer therapies.

The results of the cell-based apoptosis assay were corroborated by the histological findings, which showed no change in the positive control group or the group treated with AMS049. In contrast, the livers of animals treated with cyclophosphamide and AMS049 exhibited cytoplasmic rarefaction and eosinophilia, findings characteristic of apoptotic events [63]. In these cases, the cell-based assays showed high rates of apoptotic cells. The high frequency of apoptosis also observed in the kidneys might be related to blood vessel congestion and a decrease in the filtration capacity of this organ as demonstrated by the reduction in Bowman’s capsular spaces.

Hyperplastic and reactive lymphoid follicles forming a germinal center were observed in splenic tissue sections, a finding indicating immunomodulation. However, there was no significant change in the differential blood cell count. In addition, a reduction in relative spleen weight and in splenic phagocytosis levels was observed in the groups treated with cyclophosphamide alone or in combination with AMS049. These findings are in contrast to what is expected because the increase in circulating micronuclei should have increased splenic phagocytosis. These data are still difficult to understand, and further immunology studies are needed. However, a similar situation has been reported in the literature. In a study by Veiga [64], differential leukocyte counts in peripheral blood remained unchanged in alcohol-treated animals even in the presence of marked lymphoid hyperplasia in the spleen. Despite the difficulty in understanding these data, it is known that lymphocytic hypoplasia is associated with immunosuppressive factors and may therefore be induced by the chemotherapy drug. Because this did not occur, the immunomodulatory activity of AMS049 in response to cyclophosphamide may indicate a therapeutic potential that should be better evaluated.

Another type of immunostimulatory action was described by Navarro [2] for AMS35AA when this compound was administered simultaneously with cyclophosphamide, with the authors observing a significant increase in the number of lymphocytes.

Biochemical analysis revealed no alterations in AST, ALT, urea, creatinine, sodium, potassium, calcium or magnesium, indicating that the administration of the resorcinolic lipid alone or in combination with cyclophosphamide does not cause hepatic or renal damage. These findings suggest that the histological alterations observed in the liver and kidneys do not compromise the function of these organs. These alterations may be transient and could be regulated by apoptosis due to the high frequency of this event in the two organs. The absence of toxicity and the safety of AMS049 are positives for the biological effects of this lipid when compared with AMS35AA because alterations in AST suggesting hepatotoxicity have been described for the latter [2]. This gain suggests a beneficial effect and greater safety if this lipid is indicated for therapeutic application.

Other studies also demonstrated that the incorporation of this class of lipids into the membrane increases the efficacy and reduces the side effects of chemotherapy drugs because these drugs are transported and released by liposomes whose formation is induced by the administration of phenolic lipids [44, 65].

## Conclusion

The present results permit the inference that AMS049 (compound 3) has therapeutic potential because it does not cause genetic or biochemical changes under the experimental conditions tested and tissue alterations appear to be transient. In addition, the lipid potentiated the mutagenic effect of cyclophosphamide, which may have resulted in an increase in apoptosis. The immunomodulatory activity of AMS049 should also be highlighted. Taken together, the results suggest that AMS049 can be indicated as an important chemotherapeutic adjuvant.

## Additional files

Additional file 1: Figure AF1. ^1^H NMR spectrum for compound **2** (300 MHz, CDCl_3_).
Additional file 2: Figure AF2. ^13^C NMR spectrum for compound **2** (75 MHz, CDCl_3_).
Additional file 3: Figure AF3. ^1^H NMR spectrum for compound **3** (300 MHz, Acetone-*d*_6_).
Additional file 4: Figure AF4. ^13^C NMR spectrum for compound **3** (75 MHz, Acetone-*d*_6_).
Additional file 5: Figure AF5. DEPT-135 spectrum for compound **3** (Acetone-*d*_6_).

## Abbreviations

AMS35AA: 3-Heptyl-3,4,6-trimethoxy-3*H*-isobenzofuran-1-one
AMS049: 3-Heptyl-4,6-dihydroxy-3*H*-isobenzofuran-1-one
P.A.: Pro Analysis
TMS: Tetramethylsilane
IR: Infrared
AcOEt: Ethyl acetate
EI-MS: Electron impact mass spectrometry
AST: Aspartate aminotransferase
ALT: Alanine aminotransferase
%DR: Percentage of damage reduction
%DI: Percentage of damage increase
SE: Standard error of the average
LMP: Low melting point
TLC: Thin layer chromatography
bw: Body weight
*ip*: Intraperitoneally
HSQC: Heteronuclear single quantum coherence spectroscopy
HMBC: Heteronuclear multiple-bond correlation spectroscopy
FTIR: Fourier transform infrared spectroscopy
ESI-FT-ICR-MS: Electrospray ionization Fourier transform ion cyclotron resonance mass spectrometry
HPLC: High-performance liquid chromatography
FGI: Functional group interconversion

## Footnotes

1 DBE (double bond equivalent) = c – h/2 + n/2 + 1, where c, h, and n are the number of carbon, hydrogen, and nitrogen atoms in the molecular formula, respectively.
