# Supplementary figures and images for: A novel cytosporone 3-Heptyl-4,6-dihydroxy-3H-isobenzofuran-1-one: synthesis; toxicological, apoptotic and immunomodulatory properties; and potentiation of mutagenic damage
Source: BMC Cancer. 2015 Jul 31;15:561. doi: 10.1186/s12885-015-1532-2 (PMC4520062; doi:10.1186/s12885-015-1532-2)

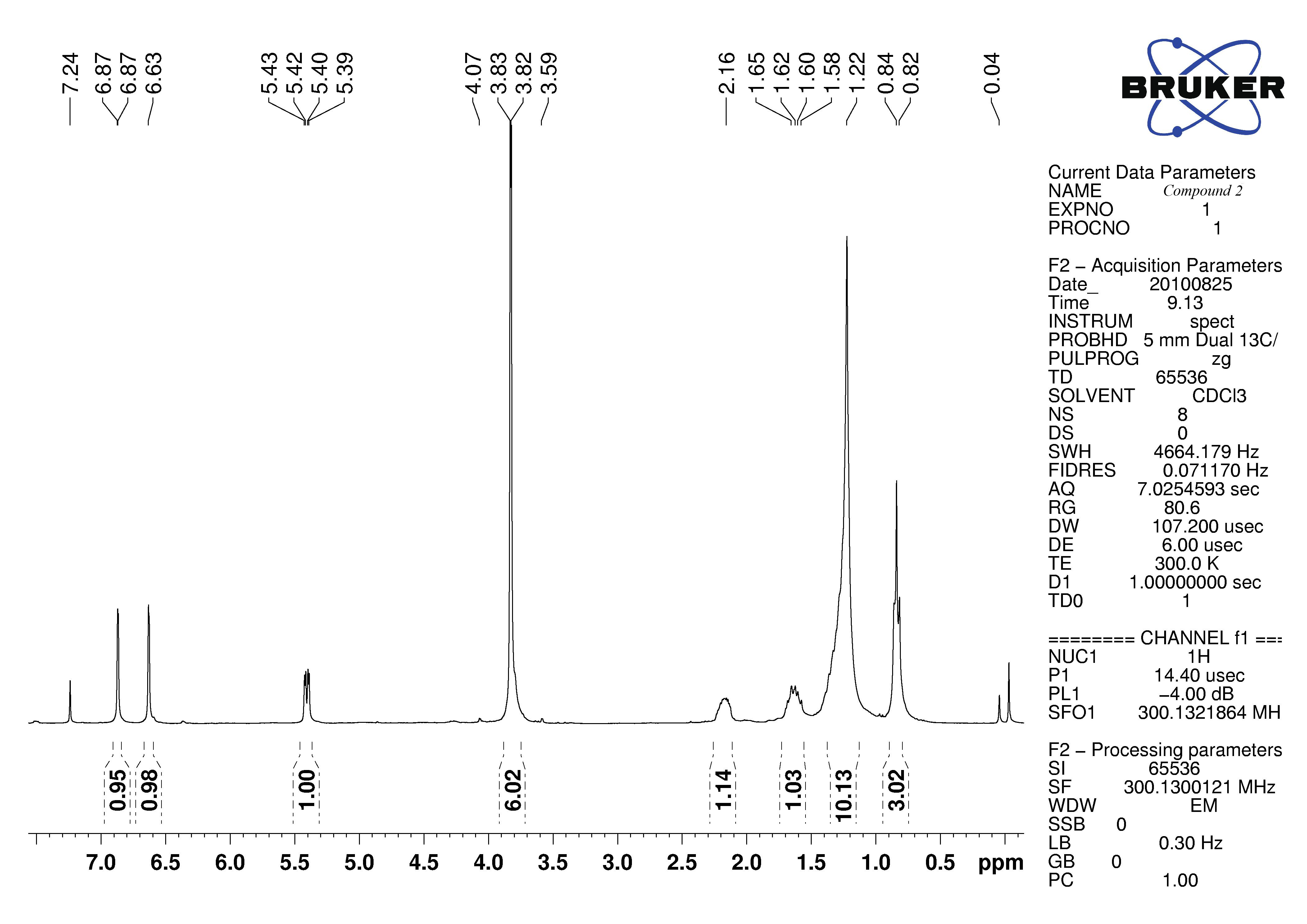

Supplement: Additional file 1: Figure AF1. — 1H NMR spectrum for compound 2 (300 MHz, CDCl3). [file 12885_2015_1532_MOESM1_ESM.jpg]

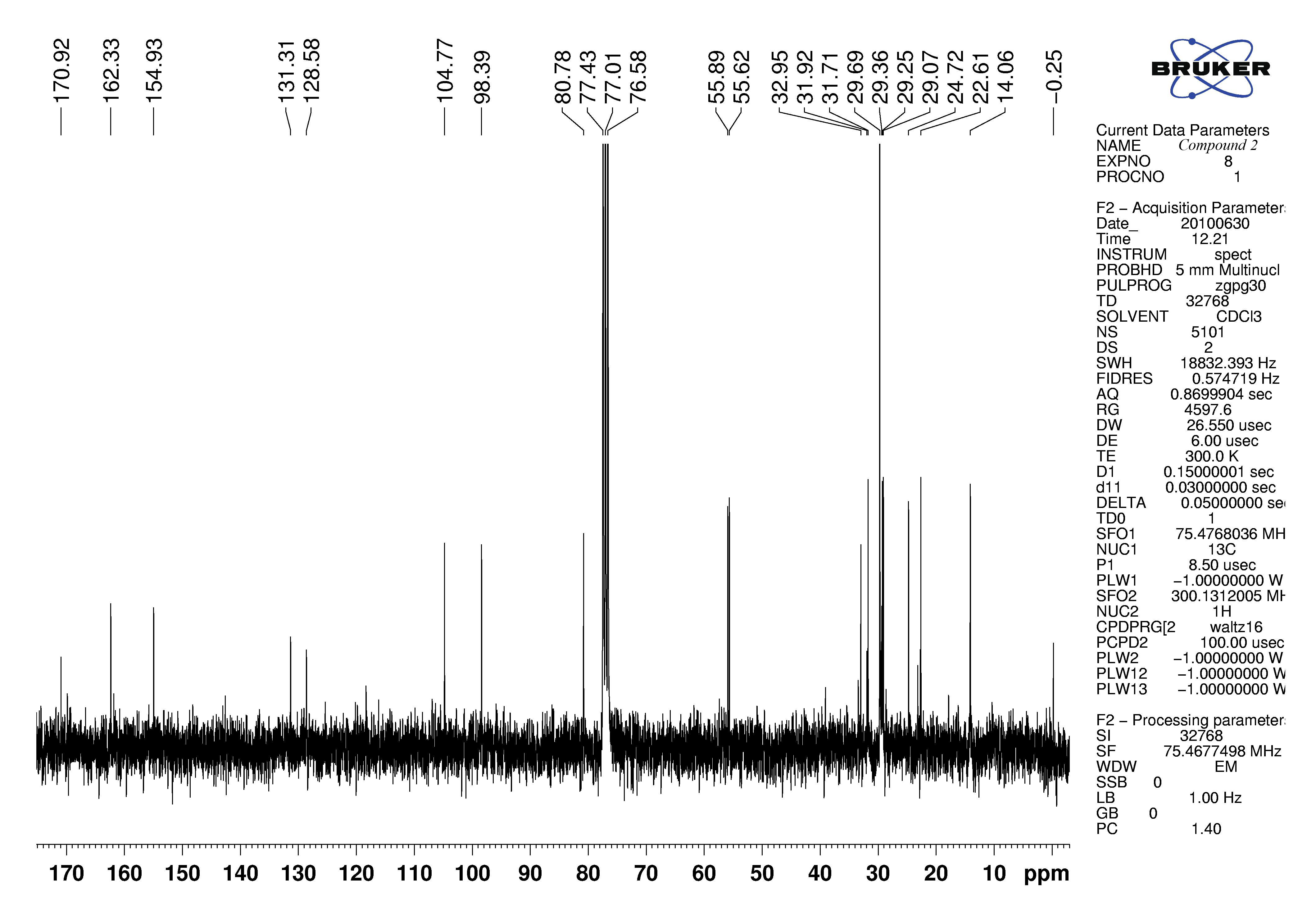

Supplement: Additional file 2: Figure AF2. — 13C NMR spectrum for compound 2 (75 MHz, CDCl3). [file 12885_2015_1532_MOESM2_ESM.jpg]

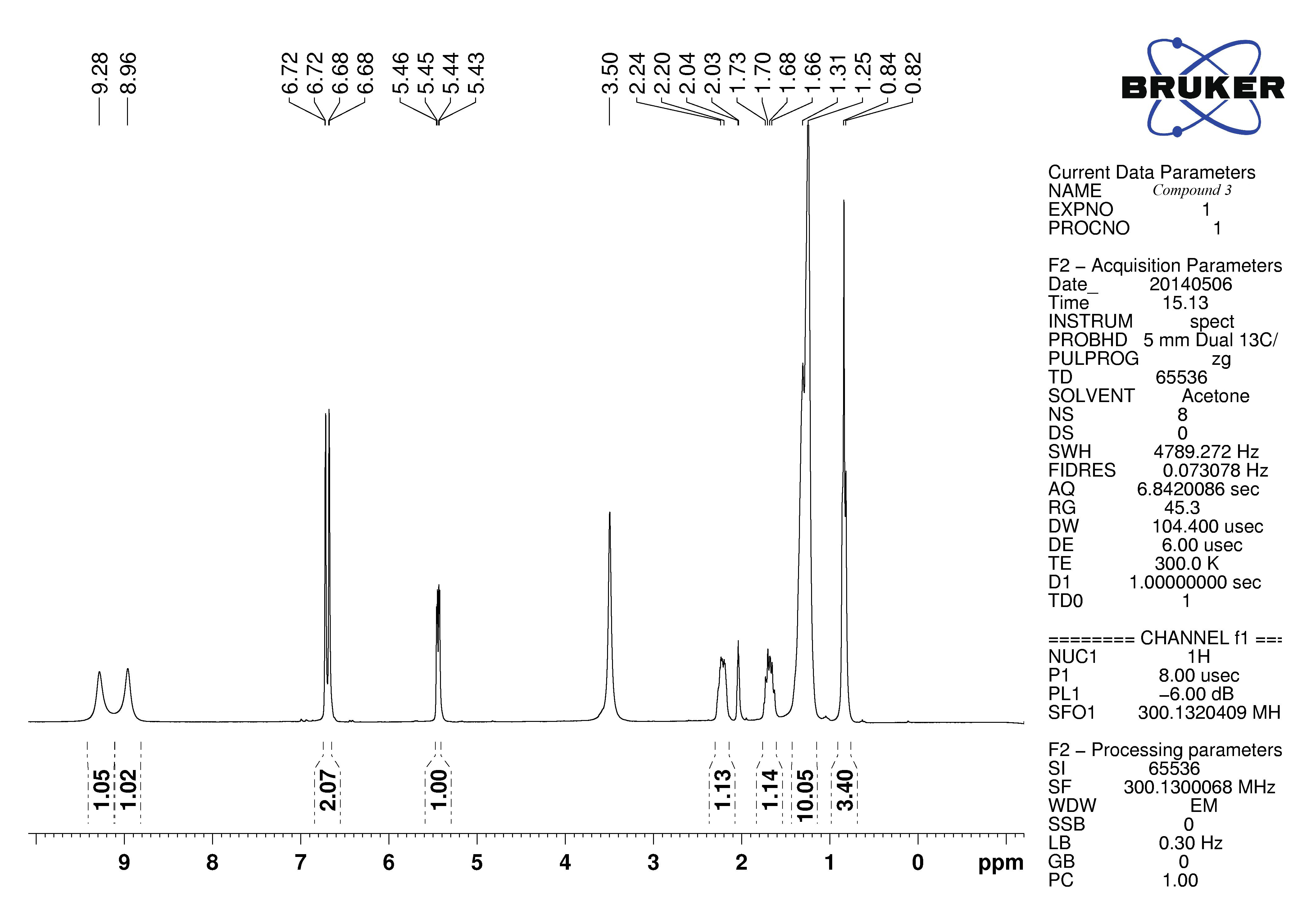

Supplement: Additional file 3: Figure AF3. — 1H NMR spectrum for compound 3 (300 MHz, Acetone-d6). [file 12885_2015_1532_MOESM3_ESM.jpg]

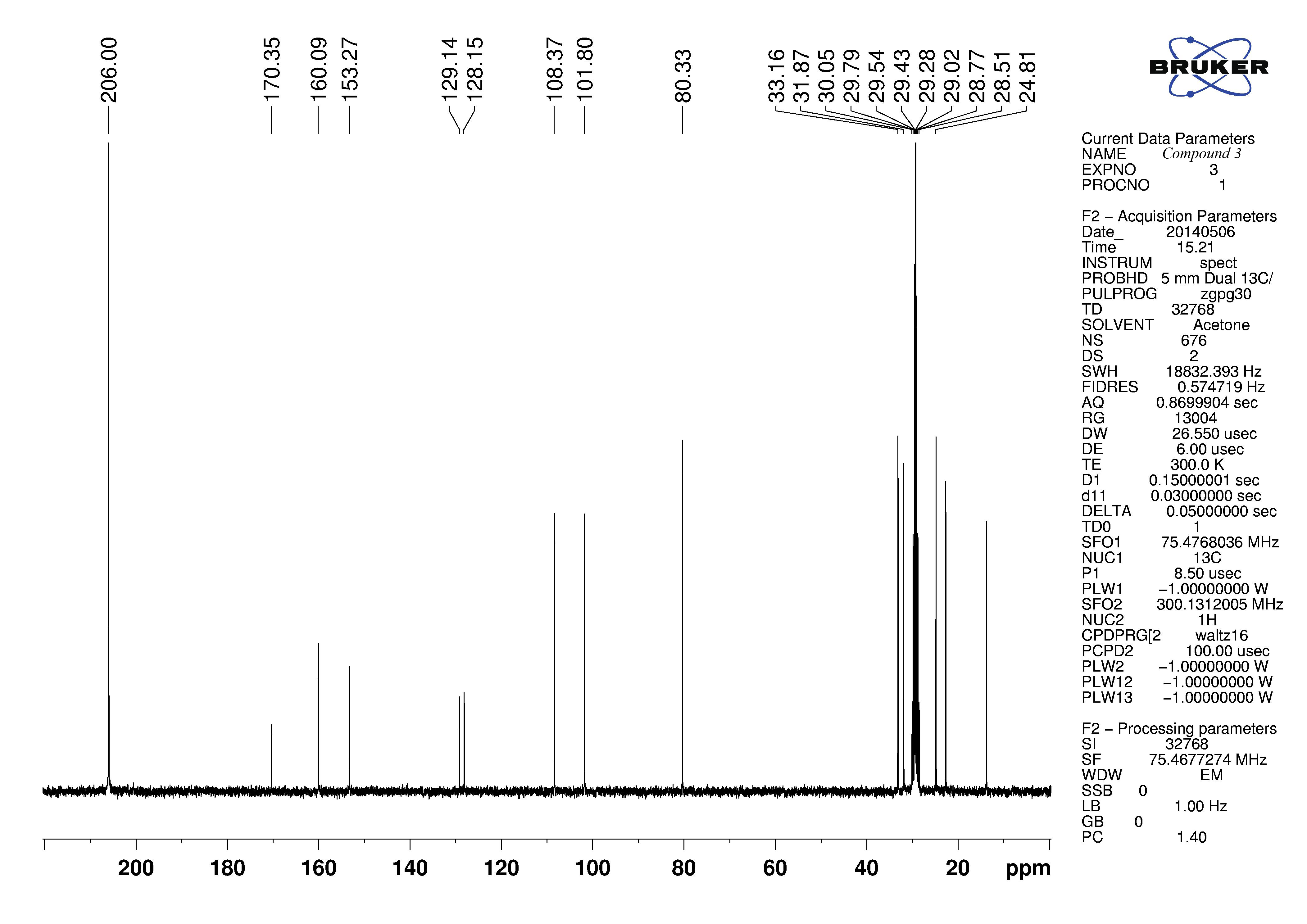

Supplement: Additional file 4: Figure AF4. — 13C NMR spectrum for compound 3 (75 MHz, Acetone-d6). [file 12885_2015_1532_MOESM4_ESM.jpg]

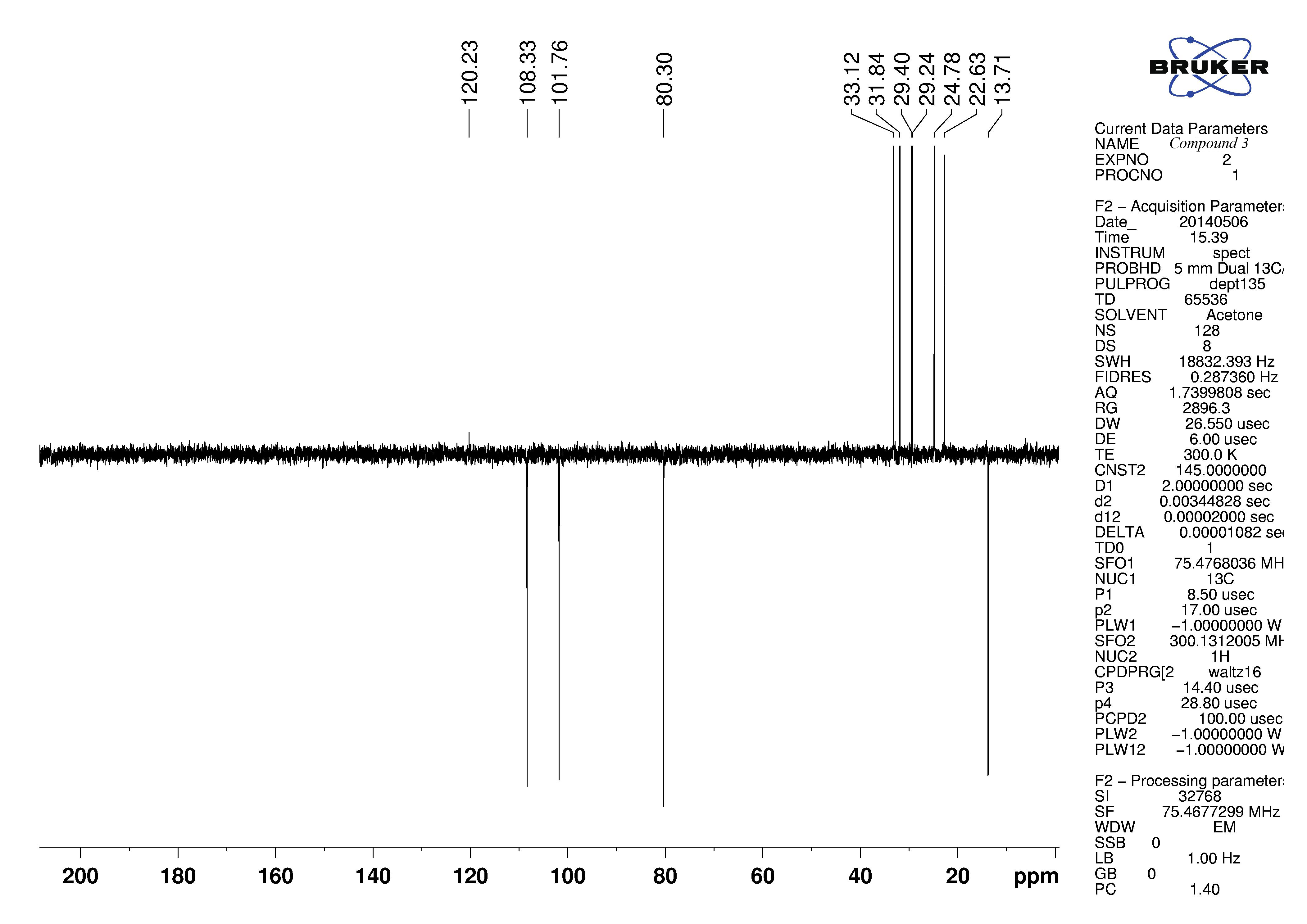

Supplement: Additional file 5: Figure AF5. — DEPT-135 spectrum for compound 3 (Acetone-d6). [file 12885_2015_1532_MOESM5_ESM.jpg]
